# Step-by-Step Transcatheter Tricuspid Valve Replacement Using the LuX-Valve Plus System

**DOI:** 10.1016/j.jaccas.2024.102699

**Published:** 2024-11-06

**Authors:** Ling-na Wong, Kevin Ka-ho Kam, Leo Kar-lok Lai, Simon Chi-ying Chow, Yat-yin Lam, Song Wan, Alex Pui-wai Lee, Kent Chak-yu So

**Affiliations:** a Division of Cardiology, Department of Medicine and Therapeutics, Prince of Wales Hospital, Chinese University of Hong Kong, Hong Kong, China; b Division of Cardiothoracic Surgery, Department of Surgery, Prince of Wales Hospital, Chinese University of Hong Kong, Hong Kong, China; c Central Medical, Hong Kong, China

**Keywords:** computed tomography, intracardiac echocardiography, transcatheter tricuspid valve replacement, transesophageal echocardiography, tricuspid regurgitation

## Abstract

The LuX-Valve Plus is a novel radial force–independent orthotopic transjugular transcatheter tricuspid valve replacement device proven to be effective in TR reduction. We describe preprocedural assessment for eligibility and procedural planning by means of computed tomography as well as procedural steps of device implantation under multimodality imaging guidance including transesophageal echocardiography (TEE), intracardiac echocardiography (ICE), and fluoroscopy: steering into the right ventricle, leaflet capture by graspers, fine valve adjustment, and septal anchor deployment. Potential pitfalls are avoided by achieving optimal alignment by means of TEE multiplanar reconstruction to steer the delivery system, and using mid-esophageal and deep gastric views of 3-dimensional TEE and supplementary ICE to visualize the graspers for leaflet capture, especially when TEE imaging is technically challenging. In the presence of paravalvular leak after valve deployment, use fine adjustment functions to optimize the result before final release.

## Graphical Abstract

**Figure:**
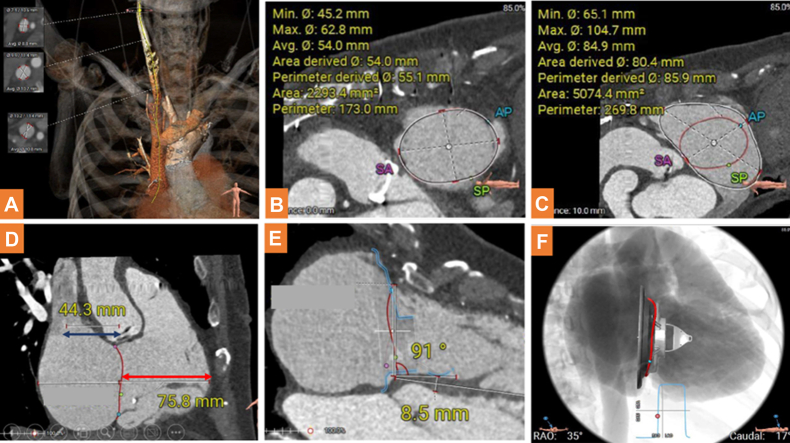

Significant tricuspid regurgitation (TR) portends a poor prognosis regardless of the underlying etiologies. The LuX-Valve Plus (Jenscare) is a novel radial force–independent orthotopic transcatheter tricuspid valve replacement (TTVR) device implanted from the internal jugular vein and has proven efficacy in substantial TR reduction.^1^ Preprocedural and intraprocedural imaging are crucial to successful TTVR. Herein, we describe preprocedural assessment for eligibility and procedural planning by means of computed tomography (CT) and summarize the key procedural steps of device implantation from 5 cases, from steering into the right ventricle (RV), leaflet capture by graspers, fine valve adjustment and septal anchor deployment under multimodality imaging guidance including 3-dimensional (3D) transesophageal echocardiography (TEE), intracardiac echocardiography (ICE), and fluoroscopy.

**Take-Home Message**

• Preprocedural and intraprocedural multimodality imaging is crucial to a successful transcatheter tricuspid valve replacement.

## Case Summary

All patients (mean age 70 years, 60% female) were deemed high risk for open heart surgery by a multidisciplinary heart team and underwent TTVR using the LuX-Valve Plus on a compassionate use basis. They were in NYHA functional class II or III. Most (80%) had torrential TR at baseline, and the TR etiologies included cardiac implantable electronic devices (CIED) related, tricuspid valve (TV) prolapse and perforation, and atrial and ventricular functional TR.

## Procedural Steps

### Preprocedural assessment

**Figure 1:**
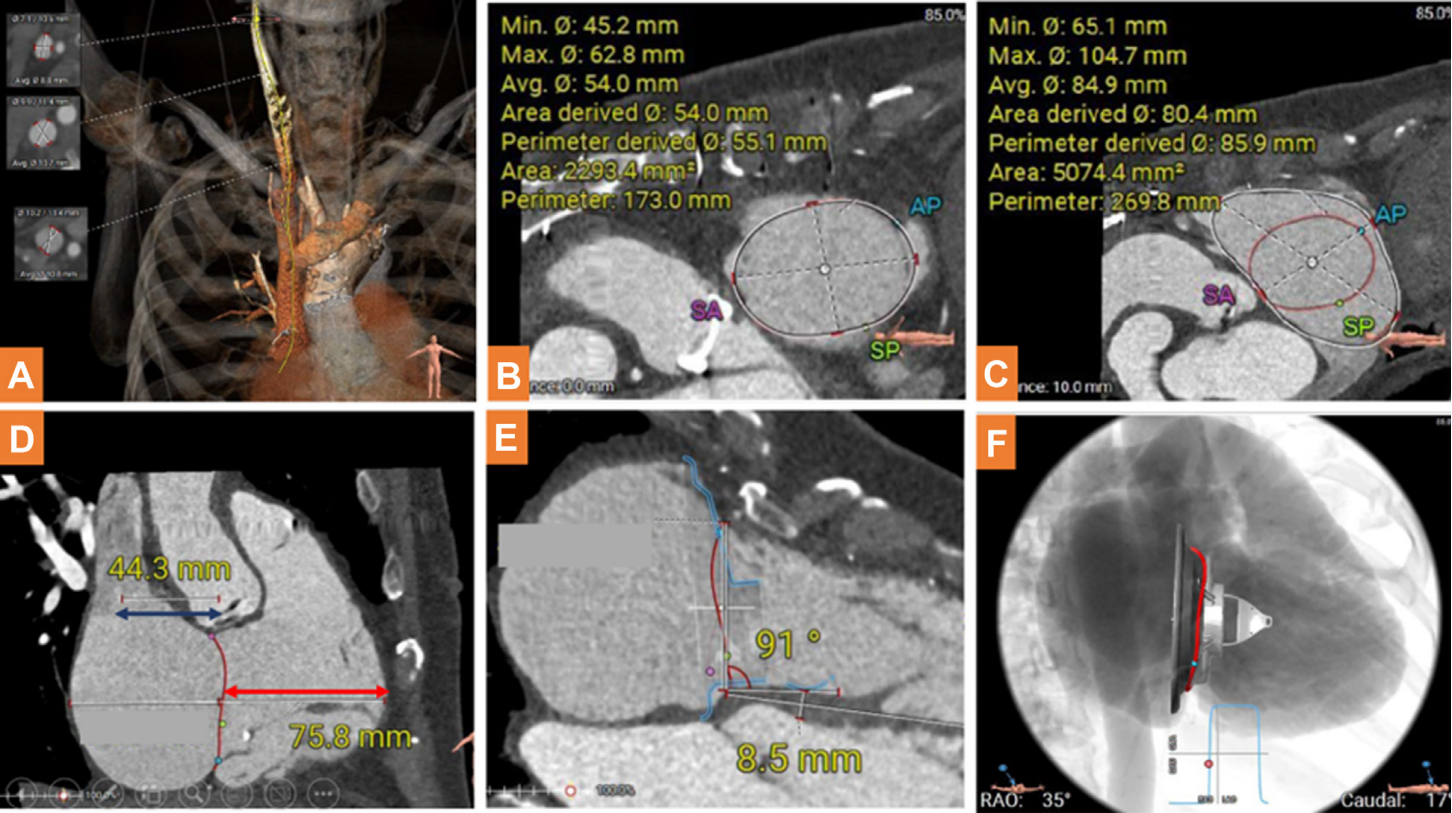
Preprocedural Computed Tomography Assessment

CT is important for case selection and procedural planning from access evaluation and device sizing to prediction of optimal deployment projections by scanning from the neck to 2 cm below the diaphragm with retrospective electrocardiographic gating (Figure 1). The diameters of the right internal jugular vein, brachiocephalic vein, and superior vena cava (≥8 mm preferred but not mandatory) were measured (Figure 1A). The TV annular dimension (perimeter-derived diameter recommended range 40-70 mm) (Figure 1B) and the dimension 1 cm above the annulus (Figure 1C) (recommended range valve size + 20 mm) were used to determine the valve size. The RV length (Figure 1D, red arrow, the distance from the center of the TV annulus to the RV free wall) and the distance between the hypothetical catheter bending point to the TV (Figure 1D, blue arrow) should be >45 mm and range from 45 mm to 70 mm. The angle between the TV annulus and the interventricular septum should be within 80°-100° for optimal valve coaxial alignment, and the interventricular septal thickness should be more than 6 mm (Figure 1E). Finally, the deployment projection was defined as one that was perpendicular to the interventricular septum (Figure 1F).

### Steering into the RV

**Figure 2:**
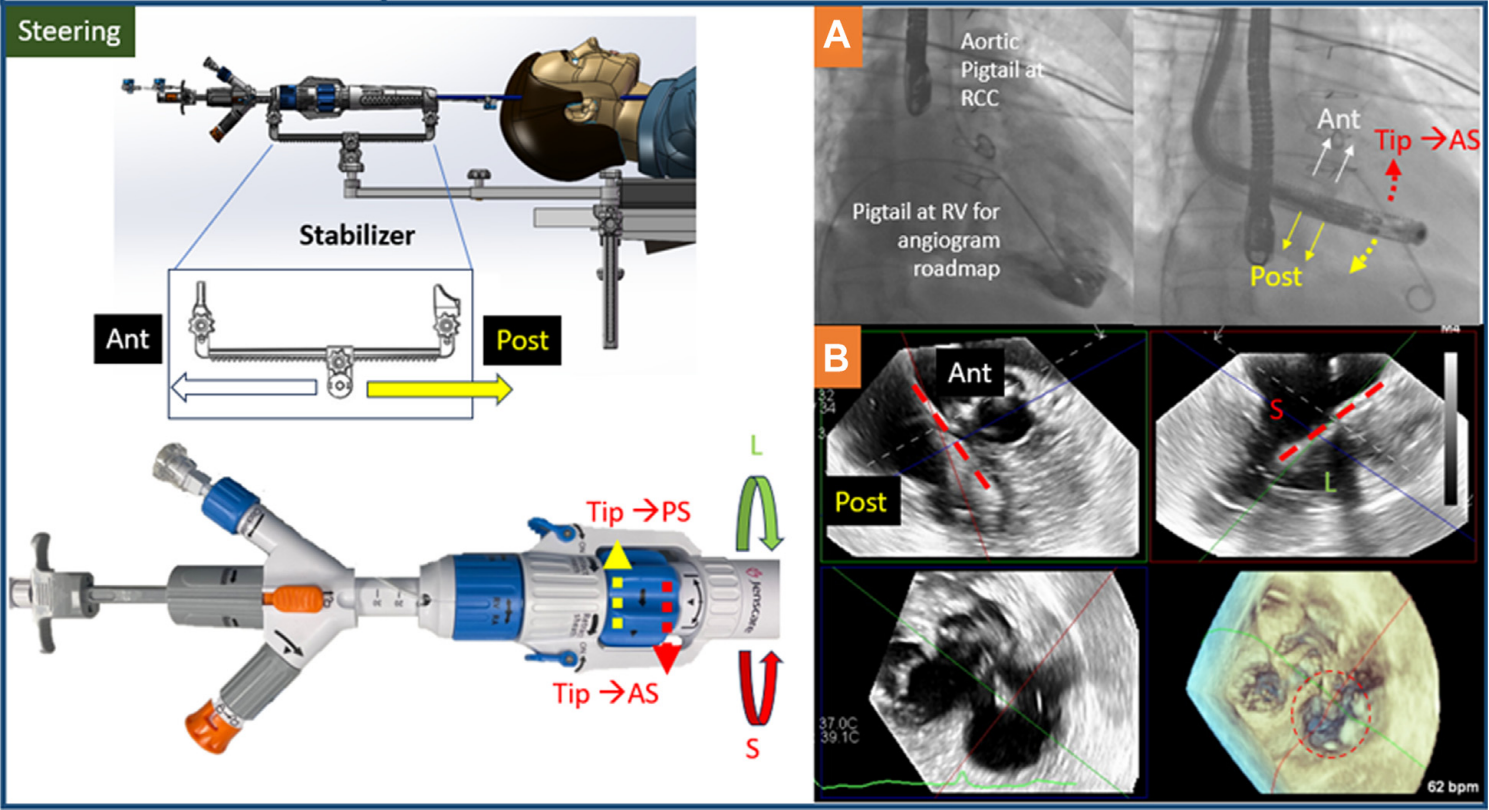
Steering Into the Right Ventricle

The LuX-Valve Plus was implanted via single jugular venous access under TEE and fluoroscopy guidance. Right internal jugular venous access was obtained under ultrasound guidance with the use of a preclose technique with 2 ProGlide devices, followed by serial dilatation to 33-F. After vascular access, the valve delivery system was inserted to mid-right atrium over a stiff wire and assembled to the sterile stabilizer (Figure 2). Although not mandatory, an aortic pigtail at the right coronary cusp landmarked the TV annulus plane and an RV pigtail can be inserted for angiogram as roadmap (Figure 2A). The delivery system was then steered into the RV without wire by gradually curving (Figure 2, Steering, red dotted arrow) and advancing the system (Figure 2, Steering, yellow arrow; Video 1). After entrance to the RV, mid-esophageal 3D TEE with multiplanar reconstruction (Video 2) was used to guide the steering of the delivery system so that it remained coaxial (Figure 2B, red dashed line) and central to the tricuspid annulus (Figure 2B, red dashed circle). By curving the system more, the tip will move more antero-septal (AS (Figure 2A, red dotted arrow), whereas un-curving will move the tip posteroseptal (Figure 2A, yellow dotted arrow). To move the system posterior, the system is advanced (Figure 2A, yellow arrow), and to move the system anterior, the system is withdrawn (Figure 2A, white arrow). To move the system septal, the system is counterclockwise rotated (Figure 2, Steering, red arrow), and to move it lateral, it is clockwise rotated (Figure 2, Steering, green arrow).

### Leaflet capture by graspers

**Figure 3:**
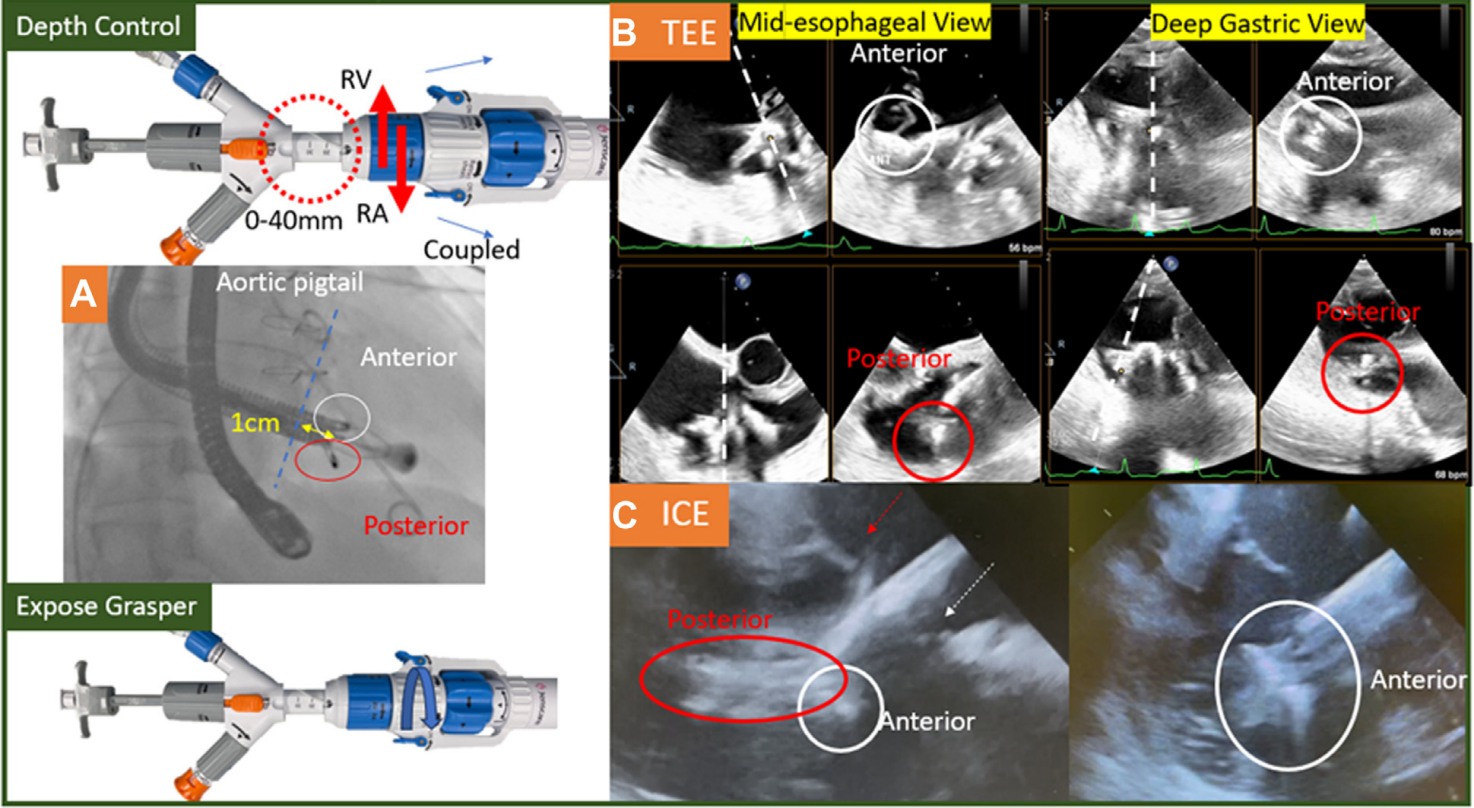
Leaflet Capture by Graspers

Before exposing the graspers (“rabbit ears”) of the LuX-Valve, the depth of the delivery system (adjustable depth 0-40 mm) was adjusted to have the base of the graspers around 1 cm ventricular to the TV annulus (Figure 3A, yellow double-sided arrow) by coupling the system and turning the blue knob (Figure 3, Depth Control, blue and red arrows; Video 3). Then the graspers were exposed by clockwise turning the white knob to unsheathe the valve ventricular portion (Figure 3, Expose Grasper, blue arrow) till the graspers were around 90 degrees perpendicular to the sheath (Figure 3A, red and white circles; Video 4). Mid-esophageal and deep gastric biplane TEE was used to identify the anterior and posterior graspers approximately 1 cm beneath the respective leaflets (Figure 3B, Video 5). ICE is not mandatory but can be used as supplemental in case of challenging TEE imaging to confirm the position of the graspers before further deployment of the device (Figures 3B and 3C).

### Fine valve adjustment

**Figure 4:**
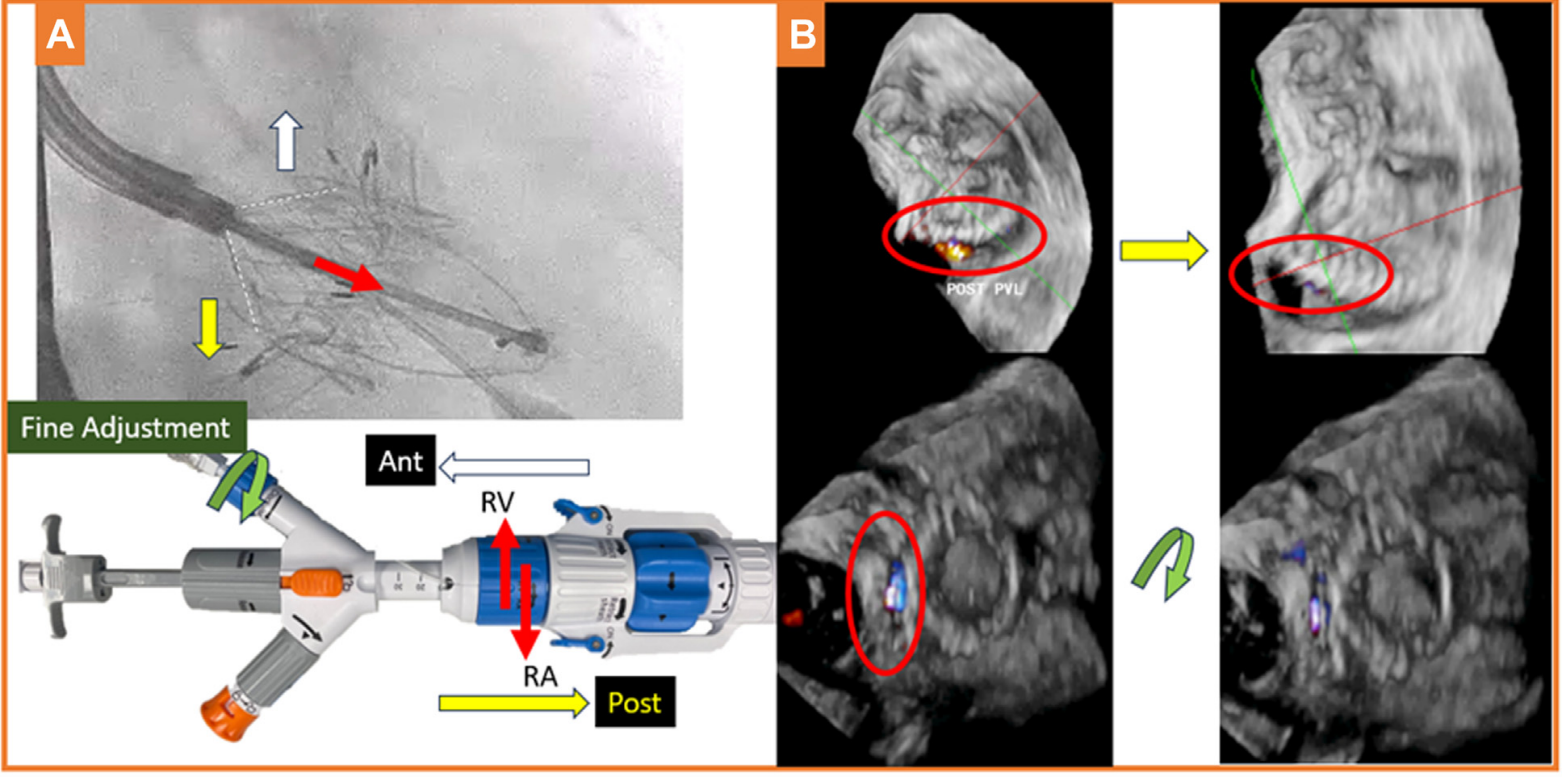
Fine Valve Adjustment

After confirming the grasper positions, the valve was deployed by further unsheathing to expose the atrial portion (Videos 6 and 7). Because the valve is still attached to the delivery system (Figure 4A, dotted white lines), fine adjustment can be made to minimize paravalvular leak (PVL). 3D color Doppler TEE was used to identify the location of PVL and guide fine adjustment. It can be performed by advancing or withdrawing the delivery system to allow better valve apposition to reduce PVL at posterior (Figure 4, yellow arrows) or anterior (Figures 4A, Fine Adjustment, white arrows). The valve axis can also be slightly adjusted by clockwise or counterclockwise rotating the small blue knob (Figure 4, Fine Adjustment, green arrow) to allow better valve conformation to the native oval TV annulus, potentially to reduce medial or lateral PVL (Figure 4B, green arrow). In case of multiple PVL, valve apposition to TV annulus might be optimized by moving the valve more ventricular or, rarely, more atrial to reduce PVL (Figure 4, Fine Adjustment, red arrows). The risk associated with valve adjustment is minimal as long as the adjustment is minor and not rough.

### Septal anchor deployment

**Figure 5:**
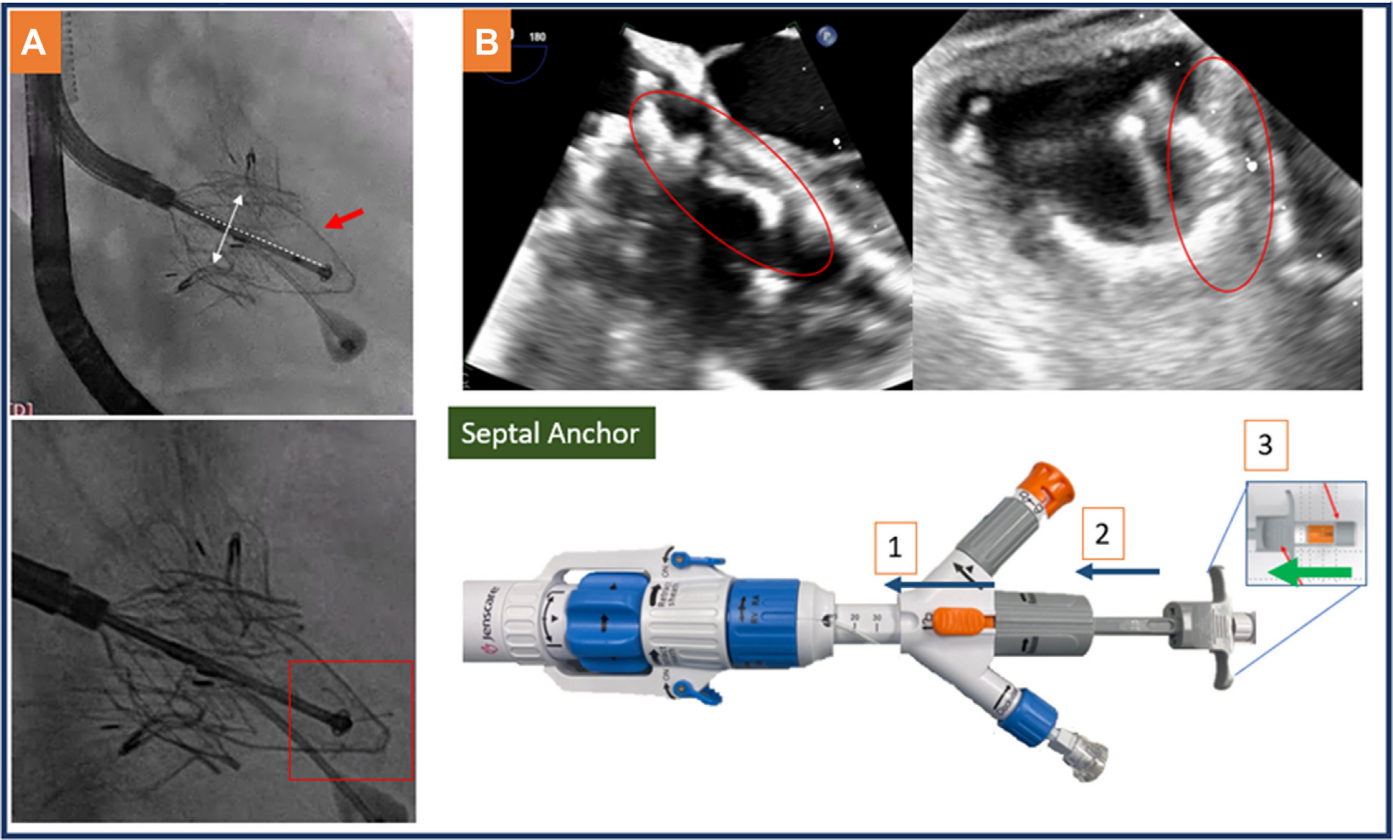
Septal Anchor Deployment

After optimizing the valve axis and apposition, the septal anchor tube was advanced by unlocking and advancing the tube (Figure 5, **Septal Anchor,** 1 and 2). The position of the septal anchor was verified with the use of fluoroscopy and TEE. On fluoroscopy, the tube (Figure 5A: dotted line) should be located at the middle of and perpendicular to the 2 graspers (Figure 5A, dotted arrow). Furthermore, optimal contact of the septal anchor plate (Figures 5A, red arrow, and 5B, red circle) and the ventricular septum was confirmed by means of TEE at mid-esophageal and deep gastric views (Video 8). Then the 3 septal anchor “hooks” are fired by advancing the back pusher on the system (Figure 5, Septal Anchor, 3, green arrow), which can be visualized on fluoroscopy (Figure 5A, red square) (Video 9). Then the valve was fully released (Video 10), and the system was gradually uncurved and withdrawn.

## Potential Pitfalls

Achieve optimal alignment by using TEE multiplanar reconstruction to steer the delivery system. Use mid-esophageal and deep gastric views of 3D TEE and supplemental ICE to visualize the graspers for leaflet capture, especially when TEE imaging is technically challenging. In the presence of PVL after valve deployment, use fine adjustment functions to optimize the result before final release.

## Clinical Follow-Up

All patients underwent successful implantations of the LuX-Valve Plus without mortality or major complications including stroke, myocardial infarction, major bleeding, conversion to open heart surgery, or conduction abnormalities requiring pacemaker implantation. Self-limiting thrombocytopenia was observed in all patients, reaching a median nadir platelet count of 63 × 10^9^/L between days 2 and 4 after TTVR. The median length of stay in hospital was 11.5 days, and all patients were discharged on warfarin.

All patients were in NYHA functional class I and II by 6- to 9-month follow up. Post-device transthoracic echocardiography showed substantial TR reduction to mild to none paravalvular TR and trace to none transvalvular TR (Video 11).

## Conclusions

TTVR using the LuX-Valve Plus to treat TR of different etiologies is feasible with preprocedural case selection. The learning curve for LuX-Valve Plus is not steep, particularly for operators and imagers experienced in tricuspid transcatheter edge-to-edge repair. Therefore, detailed understanding of the procedural steps under imaging guidance is key to outcome optimization.

## Funding Support and Author Disclosures

Dr So is a clinical proctor for Abbott, Boston Scientific, Edwards Lifescience, and Medtronic. Dr Lee is a speaker and consultant for Abbott Structural and Philips Healthcare. All other authors have reported that they have no relationships relevant to the contents of this paper to disclose.

## Appendix

Video 1 The delivery system was steered into the right ventricle without wire by gradually curving and advancing the system.

Video 2 Mid-esophageal 3-dimensional transesophageal echocardiography with multiplanar reconstruction was used to guide the fine tuning of the delivery system to ensure the system coaxially entered the tricuspid valve at its center.

Video 3 The delivery system was coaxially advanced more ventricular to ensure the base of the graspers was located ∼1 cm below the tricuspid annulus.

Video 4 The graspers were exposed by unsheathing ventricular portion of the valve until the graspers were around 90° perpendicular to the sheath.

Video 5 Mid-esophageal transesophageal echocardiography, showing the anterior and posterior graspers (“rabbit ears”), which were beneath the anterior and posterior leaflets, respectively.

Video 6 After confirming the graspers’ position on TEE, the valve was deployed by further unsheathing to expose the atrial portion.

Video 7 The atrial skirt was deployed under fluoroscopic guidance.

Video 8 Optimal contact of the septal anchor plate and the ventricular septum was confirmed with transesophageal echocardiography at mid-esophageal view.

Video 9 The 3 septal anchor “hooks” were fired to catch the ventricular septum.

Video 10 After valve deployment, 3-dimensional transesophageal echocardiography showed good valve stability and no significant transvalvular or paravalvular leak.

Video 11 TTE showed LuX Valve Plus in situ with mild PVL at septal (pacemaker lead position) and trace transvalvular TR.
